# Supplementary material for: The Efficacy of Daily Salmon Oil for Adult Type 2 Asthma: An Exploratory Randomized Double-Blind Trial
Source: Mar Drugs. 2025 Aug 15;23(8):328. doi: 10.3390/md23080328 (PMC12387695; doi:10.3390/md23080328)
Supplement: Supplementary file 1 [file marinedrugs-23-00328-s001.zip › marinedrugs-3729431-supplementary.pdf]

sensors

MDPI

Supplementary figure and table

Paper draft The efficacy of daily salmon oil for adult Th2 asthma: An ex-ploratory randomized double-blind trial

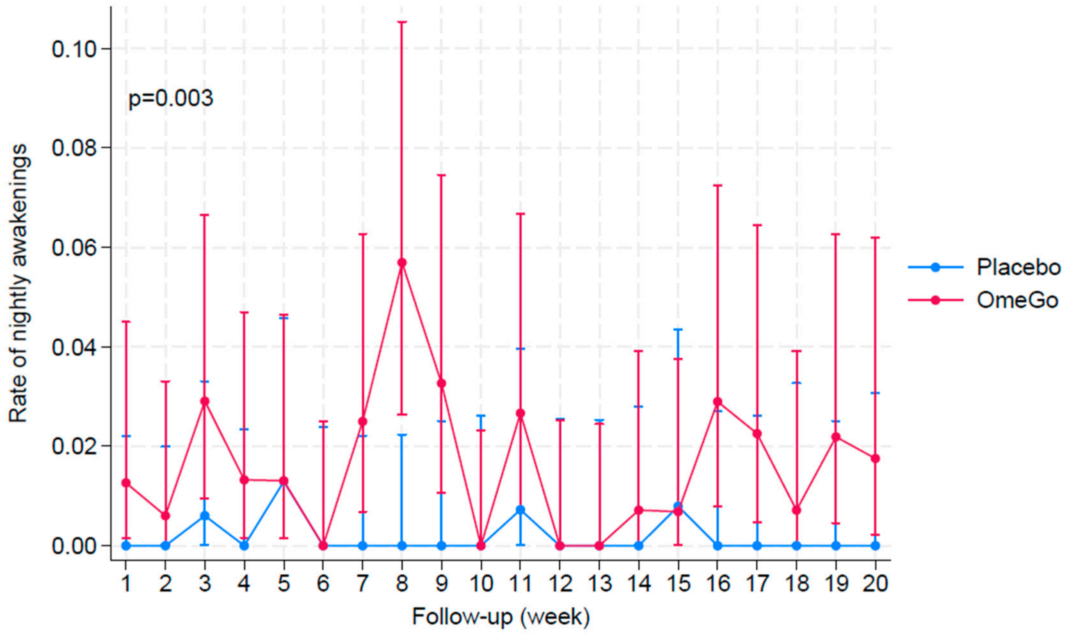

Supplementary Figure S1: Nighttime awakenings with rescue medication. The rate of registered nighttime awakenings with use of rescue medication per week. This rate is calculated as the cumulative number of nighttime awakenings divided by the total number of person-days for each week.

Supplementary Table S1: Reported gastrointestinal (GI) symptoms

	**OmeGO (N=33)**	**Causal relation to in-tervention of total number**		**Placebo (N=33)**	**Causal relation to intervention of total number**		**p-value***
**Reported gastrointestinal symptoms**		**Unlikely**	**Possible**		**Unlikely**	**Possible**	
Stomach Pain	2	0	2	7	2	5	0.073
Diarrhea	0			3	3	0	0.076
Regurgitation	4	0	4	2	0	2	0.392
Nausea	0			3	1	2	0.076
General discomfort	3	0	3	7	1	6	0.170
Obstipation	2	1	1	5	4	1	0.230

Reported gastrointestinal (GI) symptoms that do not affect daily living. *Chi-square test for differences proportions.

Supplementary Table S2: Certificate of analysis

Certificate of analysis

Author:	Version:	Approved by:	Approval date:
Henriette Heggdal	002	Angelika Florvaag	14.06.2020

**Product name:**	OmeGo®	**Production batch: so 21001**
**Product number:**	SO-01-H	**Date of production: 15.01.21**
**Country of origin:**	Norway	**Expiry date: 15.01.25**

Parameter	Result	Specification	Method
Color	Orange (salmon red)	Orange (salmon red)	Visual
Moisture	0,19	< 1%	Journal of AOAC International 93(3), 2010, p 825-832
Odour	Fresh salmon	Fresh salmon	Organoleptic
FFA	0,55	< 1 %	CDR Foodlab fat
Anisidine Value	9,85	≤ 10	CDR Foodlab fat
Peroxide Value	2,12	≤ 5 meq/kg	CDR Foodlab fat
Astaxanthin	8,77	≥ 6 µg/g	DSM Ver. 1.5 2009
Enterobacteriaceae	<10	< 300 CFU/g	AOAC 2003.01
Salmonella	Absent/25 g	Absent/25 g	AOAC 2016.01
Saturated Fatty Acids	15,0	≤ 17,0 %	Ph. Eur 2.4.29
Monounsaturated Fatty Acids	49,8	≤ 56,0 %	Ph. Eur 2.4.29
Polyunsaturated Fatty Acids	33,2	≥ 27,0 %	Ph. Eur 2.4.29
Total Omega-3 Acids	17,4	≥ 14,0 %	Ph. Eur 2.4.29
Total Omega-6 Acids	15,0	≥ 11,0 %	Ph. Eur 2.4.29
EPA	3,2	≥ 2,0 %	Ph. Eur 2.4.29
DHA	4,8	≥ 3,0 %	Ph. Eur 2.4.29
DPA	1,5	≥ 1,0 %	Ph. Eur 2.4.29
Arsenic (inorganic)[1]	<0,1	< 0.1 mg/kg	HG-AAS §64 LFGB L 25.06-1 (2008-12), mod.
Cadmium[1]	<0,01	< 0.1 mg/kg	EN ISO 15763 (2010)
Mercury[1]	<0,005	< 0.1 mg/kg	EN ISO 15763 (2010)
Lead[1]	<0,05	< 0.1 mg/kg	EN ISO 15763 (2010)
NDL-PCB[1]	20,3	< 200 µg/kg	GC-MS/MS
WHO-PCDD/F-TEQ[1]	0,499	< 1.75 ng WHO-PCDD/F-TEQ/kg	GC-MS/MS
WHO-PCB-TEQ[1]	0,913	< 3.0 ng WHO-PCB-TEQ/kg	GC-MS/MS
WHO-PCDD/F-PCB-TEQ[1]	1,41	< 4.0 ng WHO-PCDD/F-PCB-TEQ/kg	GC-MS/MS

1) Measured and reported every quarter.

Supplementary Table S3: Study flow chart

	**Screening/Inclusion/ Baseline**		**Treatment duration ±1 week**				**End of study visit**	
	**Screening/ Inclusion**	**Baseline T0 Day 0**	**T1 4 w**	**T2 8 w**	**T3 12 w**	**T4 16 w**	**End of treatment visit T5 20 w**	**T6 24 w**
Recruitment	x							
Inclusion/Exclusion	x							
Signed Informed consent	x							
Randomization		x						

	Screening/Inclusion/ Baseline		Treatment duration ±1 week				End of study visit	
	Screening/ Inclusion	Baseline T0 Day 0	T1 4 w	T2 8 w	T3 12 w	T4 16 w	End of treatment visit T5 20 w	T6 24 w
Delivery of Investigation product (CARDIO® or placebo)		x	x	x	x	x		
PEF measurement		x					x	
Vital signs [1)]		x					x	
Blood samples [2)]	x	x					x	
FEV_1 (spirometry)		x					x	x
Adverse event			x	x	x	x	x	x
Record of concomitant medication	x	x	x	x	x	x	x	
FeNO		x					x	x
Biobank [3)]	x	x					x	

1. Weight, height, body mass index (BMI), waist circumference, Blood pressure, Heart rate, Respiratory rate, arterial oxygen saturation (SaO2), Pulmonary, heart and abdominal check
2. Screening eosinophils. Baseline - Eosinophil, white blood cell count (WBC), C-reactive protein (CRP), pro-inflammatory mediators and cytokine. Safety parameters: Hemoglobin (Hb), liver function tests, kidney function tests. Capillary Omega-3 Index
3. Faeces and serum stored in -80°C.

Supplementary Table S4: Composite outcome score (COS) of moderate events

Composite variable	Definition of exacerbation	Time of measurement.	Two consecutive days of composite outcome score (COS) of 1
Peak expiratory flow (PEF)	≥20% reduction from baseline	Morning and evening	No: 0<br>Yes:1
Reliever user of SABA	Increase of dose rate of ≥ 4 puffs/day from baseline	Evening	No: 0<br>Yes:1
Day time Symptoms: Questionnaire	Increase from baseline of 1 point in 2 of 5 different questions in the questionnaire	Evening	No: 0<br>Yes:1
Night-time awakenings Questionnaire	Nocturnal awakening(s) from baseline due to asthma, requiring use of SABA	Morning	No: 0<br>Yes:1

Supplementary Table S5: Daily questions in diary in relation to asthma symptoms (PROM) and use of rescue medication

PROM Morning answers	Answers code/point
**Q1: How were your asthma symptoms during the last night?**	
No symptoms	0
Mild: symptoms not causing awakening	1
Moderate: discomfort enough to cause awakening	2

Severe: causing awakening for most of the night / did not allow sleep at all	3
**Follow-up question to Q1: If you have moderate or severe symptoms. Did you wake up last night due to asthma with rescue medicine intake?**	
Yes	1
No	0

PROM Evening answers	Answers code/point
**Q2: How was your cough during the daytime?**	
No cough	0
Mild: aware of cough which can be easily tolerated	1
Moderate: discomfort enough to cause interference with daily activity	2
Severe: incapacitating with inability to work/take part in usual activity	3
**Q3: How was your wheezing during the daytime?**	
No wheezing	0
Mild: aware of wheezing which can be easily tolerated	1
Moderate: discomfort enough to cause interference with daily activity	2
Severe: incapacitating with inability to work/take part in usual activity	3
**Q4: How was your chest tightness during the daytime?**	
No chest tightness	0
Mild: aware of chest tightness which can be easily tolerated	1
Moderate: discomfort enough to cause interference with daily activity	2
Severe: incapacitating with inability to work/take part in usual activity	3
**Q5: How was your breathlessness during the daytime?**	
No breathlessness	0
Mild: aware of breathlessness which can be easily tolerated	1
Moderate: discomfort enough to cause interference with daily activity	2
Severe: incapacitating with inability to work/take part in usual activity	3

Use of rescue medication	Answers code/point
**How many puffs of e.g. Ventolin or similar medicine (rescue medicine in case of acute deterioration) have you taken in the last 24 hours?**	
No puff	0
Yes, number of puffs ……	1

Supplementary Table S6: GINA asthma symptom control

Level of asthma symptom control				
**In the past 4 weeks, has the patient had**		**Well controlled**	**Partly controlled**	**Uncontrolled**
Daytime asthma symptoms more than twice/week?	Yes ☐ No ☐	None of these	1–2 of these	3–4 of these
Any night waking due to asthma?	Yes ☐ No ☐			
SABA reliever for symptoms more than twice/week?	Yes ☐ No ☐			
Any activity limitations due to asthma?	Yes ☐ No ☐			
